# Crystal Growth, Structure, and Noninteracting Quantum Spins in Cyanochroite, K_2_Cu(SO_4_)_2_·6H_2_O

**DOI:** 10.1021/acsomega.1c06143

**Published:** 2022-02-02

**Authors:** Darren C. Peets, Maxim Avdeev, Marein C. Rahn, Falk Pabst, Sergey Granovsky, Markus Stötzer, Dmytro S. Inosov

**Affiliations:** † Institut für Festkörper- und Materialphysik, Technische Universität Dresden, 01069 Dresden, Germany; ‡ Australian Nuclear Science and Technology Organisation, Lucas Heights, NSW 2234, Australia; § School of Chemistry, The University of Sydney, Sydney, NSW 2006, Australia; ⊥ Anorganische Chemie II, Technische Universität Dresden, 01069 Dresden, Germany; ∥ Faculty of Physics, M.V. Lomonossow Moscow State University, Moscow 119991, Russia; ¶ Anorganische Chemie I, Technische Universität Dresden, 01069 Dresden, Germany; # Würzburg-Dresden Cluster of Excellence on Complexity and Topology in Quantum Matter−ct.qmat, Technische Universität Dresden, 01069 Dresden, Germany

## Abstract

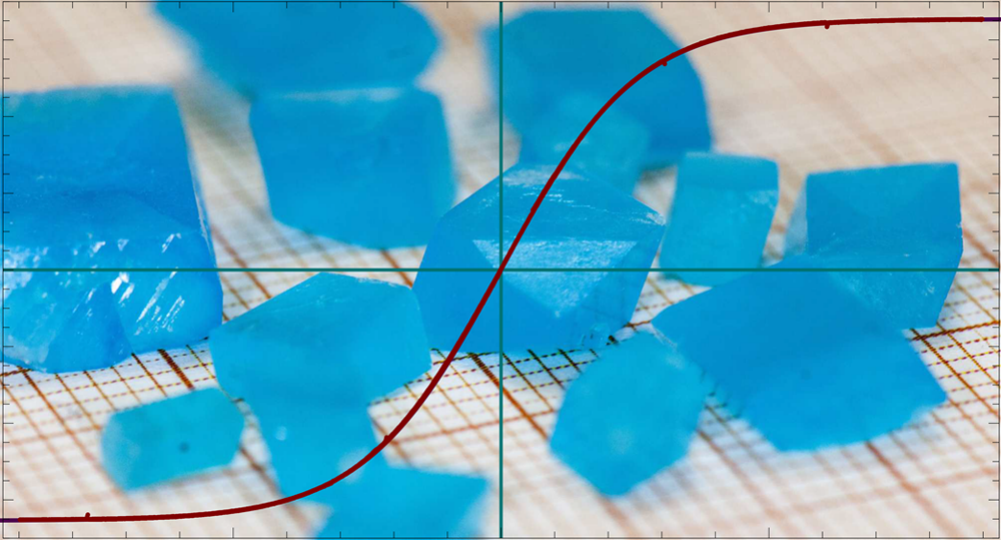

The rare mineral cyanochroite, K_2_Cu(SO_4_)_2_·6H_2_O, features isolated Cu^2+^ ions in distorted octahedral coordination, linked via a hydrogen-bond network. We have grown single crystals of cyanochroite as large as ∼0.5 cm^3^ and investigated structural and magnetic aspects of this material. The positions of hydrogen atoms deviate significantly from those reported previously based on X-ray diffraction data, whereas the magnetic response is fully consistent with free Cu^2+^ spins. The structure is not changed by deuteration. Density functional theory calculations support our refined hydrogen positions.

## 1 Introduction

Natural minerals offer a wealth of crystal structures and magnetic sublattices, and populating these sublattices with quantum spins, notably Cu^2+^, is expected to reveal exotic magnetic ground states and quantum spin dynamics.^1,2^ In a few materials, the Cu^2+^ ions are well-separated by large anions, aquo or hydroxo ligands, and alkali-metal ions, resulting in large Cu–Cu distances with long and convoluted superexchange paths. This is the case, in particular, in the Tutton salts, which have the general formula A_2_M(XO_4_)_2_·6H_2_O^a^ where A is an alkali metal or NH_4_^+^, M is a 3d transition metal, and X is sulfur or selenium, as well as in kröhnkite Na_2_Cu(SO_4_)_2_·2H_2_O^3^ and related minerals. This is expected to result in exceedingly weak magnetic interactions and in spin dynamics restricted to very low energies and temperatures.

**Figure 1:**
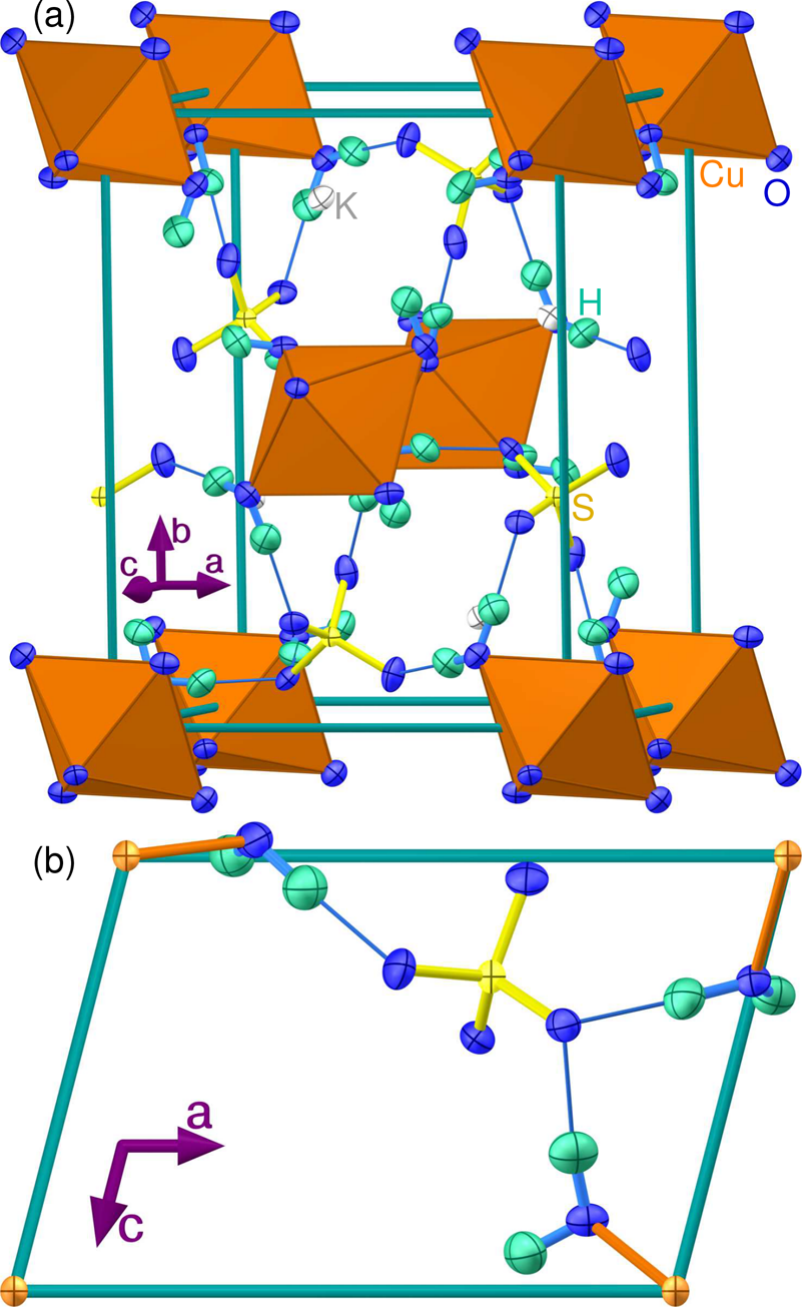
(a) Crystal structure of cyanochroite from our structure refinement at 293 K. Thinner lines indicate hydrogen bonds. (b) View along *b* of a slice showing the networks responsible for two magnetic exchange pathways.

The exchange pathways in the Tutton salts, two of which are shown in Figure 1b for cyanochroite, K_2_Cu(SO_4_)_2_·6H_2_O, pass through a minimum of five intermediate atoms via two hydrogen bonds. This would not be expected to support strong magnetic interactions, and no magnetic entropy is visible down to 2 K.^4^ The paramagnetic nature of the Tutton salts down to very low temperatures has been known for quite some time and led to these materials being well-studied decades ago by electron paramagnetic resonance (see ref (5) on cyanochroite) and for use in adiabatic demagnetization refrigeration,^6^ which led to hints of a transition around 10 mK^7^ in K_2_Cu(SO_4_)_2_·6H_2_O. The magnetic transition in cyanochroite has since been identified at 29.6 mK by ac susceptibility and specific heat measurements.^8−10^ The quest of late in condensed matter physics has been for strong electron correlations and magnetic frustration, but these families offer a useful baseline of maximally noninteracting Cu^2+^ ions.

The Tutton salts including cyanochroite, the structure of which is shown in Figure 1a, have also been investigated for use in Li and Na batteries^11^ and for a unique interplay between their hydrogen-bonding networks and their Jahn–Teller-distorted Cu coordination spheres. Although most Tutton salts form in the same crystal structure, the copper members of the family can form in two different structures, determined by a competition between cooperative Jahn–Teller distortions and the hydrogen-bonding network.^12−15^ The copper-based Tutton salts can be tuned between these structures by pressure,^16,17^ by doping, or by substitution on the Cu,^18^ X,^19^ or A site.^20^ (NH_4_)_2_Cu(SO_4_)_2_·6H_2_O is even known to adopt different structures depending on the degree of deuteration.^21,22^

We find a magnetic response in K_2_Cu(SO_4_)_2_·6H_2_O consistent with free Cu^2+^ spins at all fields and temperatures accessible to us. Our crystal structure refinement provides updated hydrogen positions, which differ significantly from earlier values extracted from X-ray diffraction,^23^ and establishes that deuteration does not significantly alter the crystal structure or magnetic response of K_2_Cu(SO_4_)_2_·6H_2_O. Our hydrogen positions will be useful for modeling the magnetic interactions, internal electric fields, and octahedral distortions.

## 2 Results and Discussion

### 2.1 Diffraction

**Figure 2:**
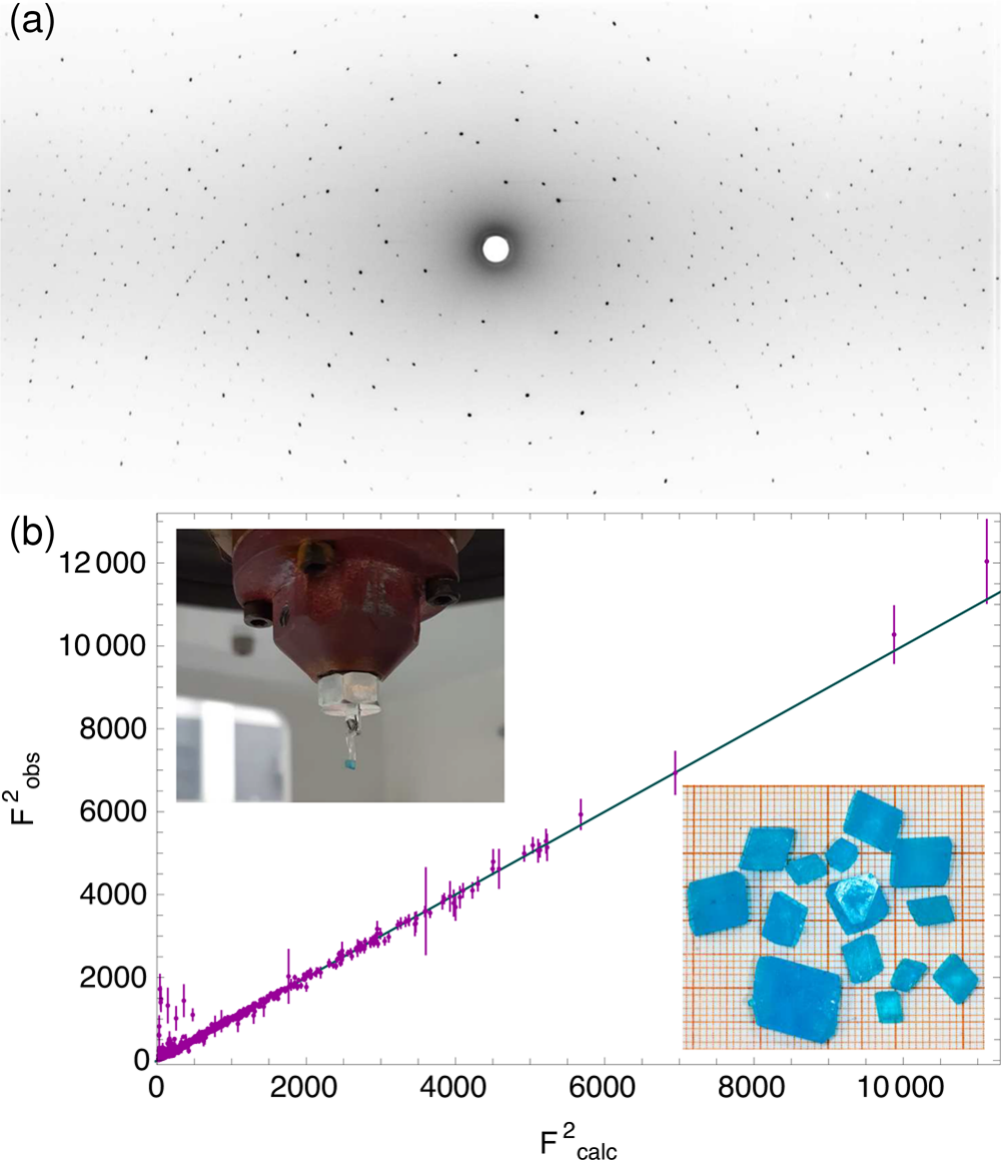
Neutron white-beam Laue diffractometry on cyanochroite. (a) Laue pattern at 293 K, showing sharp spots. (b) Comparison of observed and calculated structure factors, demonstrating the quality of the refinement. The top inset shows the crystal used, and the bottom inset shows several representative crystals on millimeter-ruled graph paper.

Neutron white-beam Laue diffraction was performed on cyanochroite at room temperature and 4 K and in multiple orientations to ensure reflection coverage. One of the Laue images for a measurement at room temperature is shown in Figure 2a, and the top inset in Figure 2b shows the crystal mounted in the beam. The corresponding |*F*^2^|_calc_ versus |*F*^2^|_obs_ plot from the refinement of these data is shown in Figure 2b. The points remain close to a unit slope, indicating that the refined structure describes the data well.

**Figure 3:**
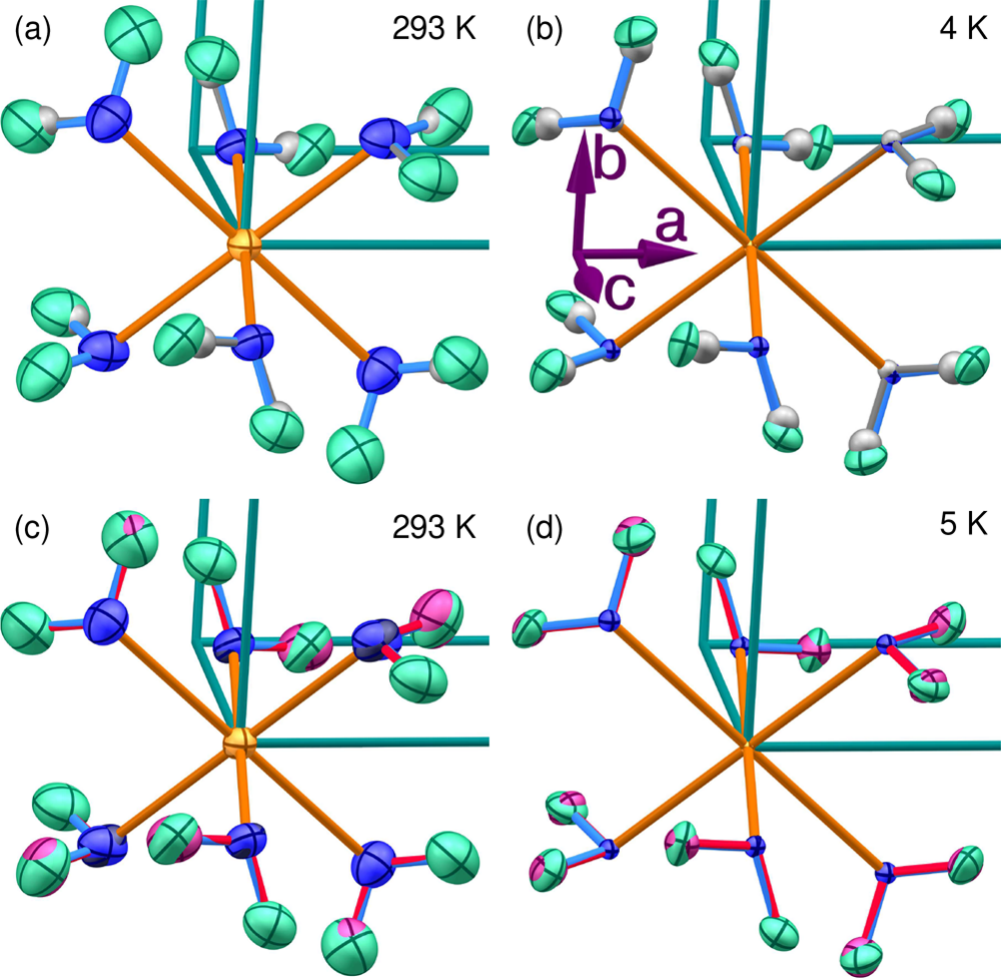
Our refined hydrogen positions (50% probability ellipsoids, green) at (a) 293 and (b) 4 K compared with the room temperature hydrogen positions (gray) of ref (23). The shifts are significant at all sites. Shifts on the oxygen sites at 4 K reflect the lower measurement temperature in our data. Blue is oxygen, and yellow is copper. Our refined deuterium positions (magenta) at (c) 293 and (d) 5 K are compared with our respective hydrogen positions. Oxygen ellipsoids for the deuterated structure are drawn darker.

The results of refinements at 293 and 4 K are summarized in Table 1. With the exception of hydrogen positions, our refined crystal structure is consistent with that previously reported, but we were additionally able to refine anisotropic thermal parameters. The hydrogen positions are significantly different from their previously reported positions,^23^ exhibiting shifts of 0.11 to 0.25 Å at room temperature. At 4 K, our atomic positions shift slightly, and the displacements relative to ref (23) range from 0.14 to 0.25 Å. The hydrogen positions refined from our data are compared with those from ref (23) in Figure 3 to more clearly demonstrate the significance of these shifts. The work of ref (23) was based on single-crystal X-ray diffraction, which is sensitive to electron density and thus has far poorer sensitivity to H than does neutron diffraction, so the hydrogen positions reported here more closely represent the location of the nuclei. This is supported by a visual examination of the shifts in hydrogen positions—the X-ray-derived hydrogen positions are all significantly closer to the covalently bonded oxygen atom, as that covalent bond is where the electron density is.

**Table 1:** Parameters of the Neutron Structure Refinement of K_2_Cu(SO_4_)_2_·6H_2_O and K_2_Cu(SO_4_)_2_·6D_2_O (“D”) in Space Group *P*2_1_/*a* (No. 14) at 293 K and Low Temperature, and the X-ray Structure Refinement of K_2_Cu(SO_4_)_2_·6D_2_O at Room Temperature^a^

temperature	293 K	4 K	293 K (D)	5 K (D)	293 K (D, XRD)
space group	*P*2_1_/*a*	*P*2_1_/*a*	*P*2_1_/*a*	*P*2_1_/*a*	*P*2_1_/*a*
*a*	9.090 Å	9.082 Å	9.085 Å	9.092 Å	9.0647(5) Å
*b*	12.121 Å	12.108 Å	12.137 Å	12.106 Å	12.0875(7) Å
*c*	6.167 Å	6.182 Å	6.162 Å	6.173 Å	6.1516(4) Å
β	104.55°	104.43°	104.36°	104.31°	104.453(5)°
unit cell volume	657.68 Å^3^	658.36 Å^3^	658.22 Å^3^	658.37 Å^3^	652.70(7) Å^3^
*Z*	2	2	2	2	2
density	2.232 g/cm^3^	2.229 g/cm^3^	2.288 g/cm^3^	2.287 g/cm^3^	2.310 1 g/cm^3^
reflections	4412	4389	4424	3271	1302
reflections, *I* > 3σ	2154	3253	1396	2348	1256
parameters	143	143	143	143	95
θ range	2.86–51.20°	3.40–51.09°	3.80–51.62°	3.98–51.21°	2.87–27.25°
coverage	0 ≤ *h* ≤ 18	0 ≤ *h* ≤ 18	0 ≤ *h* ≤ 15	0 ≤ *h* ≤ 15	–10 ≤ *h* ≤ 11
	0 ≤ *k* ≤ 24	0 ≤ *k* ≤ 24	0 ≤ *k* ≤ 26	0 ≤ *k* ≤ 26	–15 ≤ *h* ≤ 12
	–12 ≤ *l* ≤ 11	–13 ≤ *l* ≤ 11	–10 ≤ *l* ≤ 11	–10 ≤ *l* ≤ 11	–7 ≤ *l* ≤ 7
*R*, *I* > 3σ	5.41%	4.39%	6.21%	5.55%	4.51%
*wR*, *I* > 3σ	4.23%	3.95%	4.75%	4.83%	5.93%

a Lattice parameters from the neutron Laue measurements were estimated at the data reduction stage.^24^

Several deuterated crystals were also measured, by both neutron and X-ray single-crystal diffraction, to determine whether the structure changes with deuteration as it does in (NH_4_)_2_Cu(SO_4_)_2_·6H_2_O. The X-ray structure solution algorithm reproducibly converged to the (Cu, K, S, O) atomic arrangement previously reported.^19,23,25^ This measurement is not sensitive to the presence of deuterium ions due to their weak X-ray scattering amplitude, so positional parameters for D were fixed to those determined by neutron diffraction at the same temperature. Excluding D entirely produced significantly worse results, and refining these positional parameters led to insignificant improvements. Non-hydrogen structural parameters were refined using Jana2006; the refinement is summarized in Table 1. Both X-ray and neutron diffraction indicated that the structure does not change with deuteration, and the atomic positions are essentially unchanged.

In the harmonic approximation, the ratio of atomic displacement parameters *U*_eq_(H)/*U*_eq_(D) should be inversely proportional to the square root of the ratio of their masses, that is, √2 ∼ 1.4. The fact that the refined ratio is significantly lower—1.063(12) and 1.15(2) at 293 and 4 K, respectively—suggests anharmonicity and/or local static disorder. The latter is also supported by the fact that, although the thermal displacement parameters for Cu, K, S, and O decrease by a factor of ∼5 on cooling from room temperature to 4 K, those of H and D decrease only by a factor of ∼2.

### 2.2 Density Functional Theory Calculations

**Figure 4:**
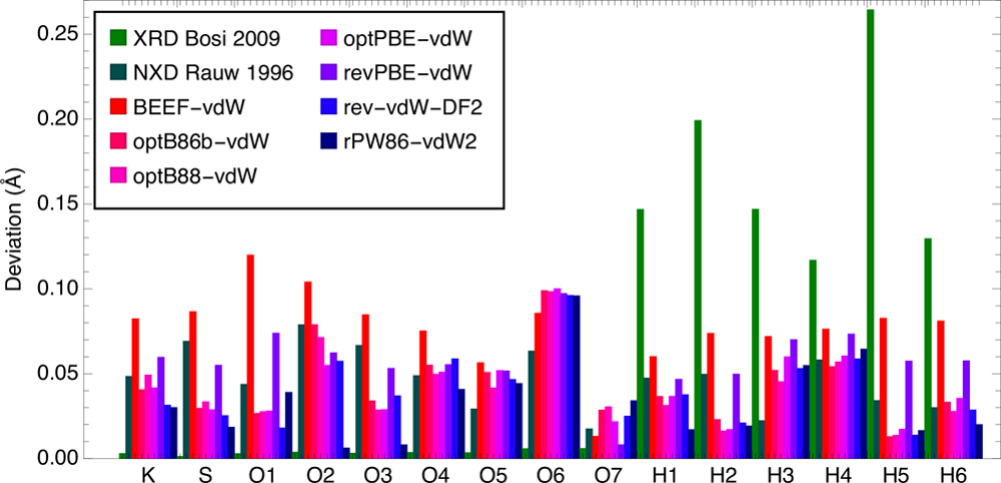
Comparison of the deviations in Å between the positions from our neutron structure refinement and the atomic positions determined by Bosi et al.,^23^ by Rauw et al.,^25^ and by DFT calculations using all available van der Waals density functionals.

Density functional theory (DFT) calculations were used to model the crystal structure of cyanochroite, using all van der Waals density corrections and functionals available in VASP.^26^ DFT is, in principle, a zero-temperature approximation, so results were compared against our 4 K results, which were collected specifically for this comparison. All calculations produce a crystal structure consistent with our refinements and previous X-ray diffraction results, but with deviations in the atomic positions. These deviations from our 4 K neutron refinement are shown in Figure 4, where the X-ray results of Bosi et al.^23^ are also included. Atomic positions here were converted to angstrom units using the experimental 4 K unit cell.

Excluding hydrogen positions, the atomic positions reported previously based on room-temperature X-ray diffraction^23^ are roughly an order of magnitude closer to our current results than those of any DFT model or the neutron single-crystal refinement under 1.4 kbar of pressure reported in ref (25), despite the very different temperature. The X-ray lattice parameters are also very close to ours (not shown), again despite the temperature difference, but here, the others are not significantly worse. The X-ray refinement fares poorly when it comes to hydrogen positions because X-ray diffraction probes electron density rather than the position of the nucleus, and the electron density around H^+^ is both extremely low and shifted by typically 0.1–0.2 Å toward the nearest anion.

Among the van der Waals functionals, BEEF-vdW consistently produced the least accurate results, whereas rPW86-vdW2 gave the best results for 8 of the 15 refineable sites. In particular, rPW86-vdW2 came far closer to the experimental O2 and O3 positions than any other functional. The O6 site seemed particularly difficult to model accurately through DFT, although interestingly, the otherwise inaccurate BEEF-vdW functional described this site better than the other functionals.

## 3 UV–Vis Spectroscopy

**Figure 5:**
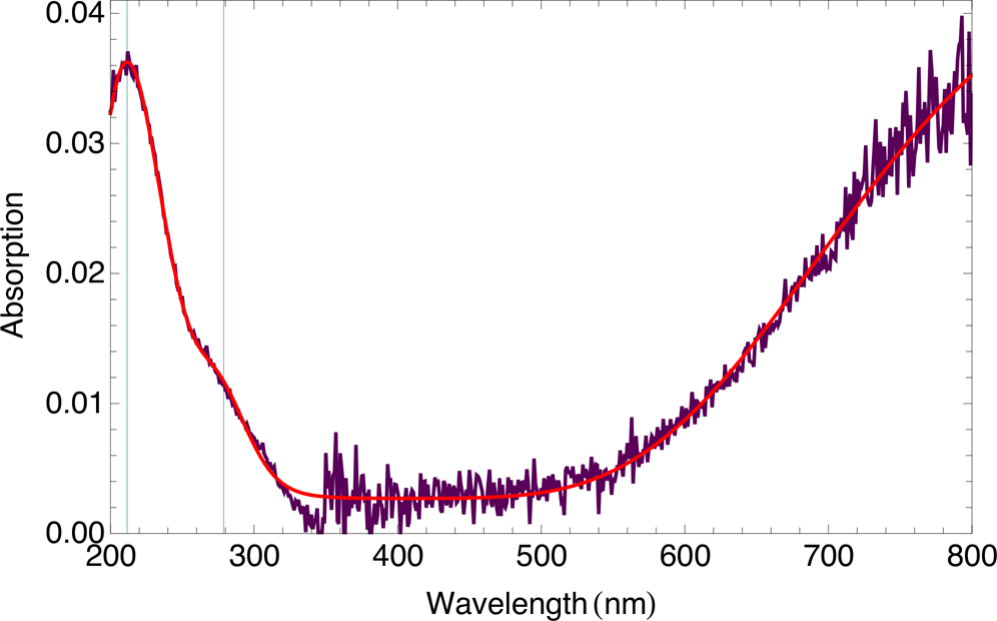
UV–vis–NIR spectrum of cyanochroite, with a fit to three Gaussians.

The absorption spectrum of K_2_Cu(SO_4_)_2_·6H_2_O in the near-infrared, visible, and ultraviolet range is shown in Figure 5. This spectrum was fit to three Gaussians in the frequency domain to obtain peak positions. The first two peaks are found at 212 and 279 nm (47 255 and 35 862 cm^–1^), whereas the third peak is in the infrared range beyond our measurement window.

In a conventional Jahn–Teller picture considering only the ligands directly bonded to Cu, the sole hole in elongated octahedrally coordinated Cu^2+^ would be expected to be in the d_*x*^2^–*y*^2^_ antibonding orbital, and excitations would only be possible into this orbital. Transitions would be possible, in order of increasing energy, from d_3*z*^2^–*r*^2^_, d_*xy*_, and the doubly degenerate d_*xz*_ and d_*yz*_. However, recent work has shown that the Cu coordination sphere in Tutton salts is best described as a *compressed* octahedron with an orthorhombic instability, where electric fields from more distant ions play a crucial role .^27^ In this picture, the d_3*z*^2^–*r*^2^_ antibonding orbital is shifted to higher energy than d_*x*^2^–*y*^2^_, and the lowest-energy transition is from d_*x*^2^–*y*^2^_ to d_3*z*^2^–*r*^2^_. This transition is not within our measurement window, and we cannot comment on the veracity of this picture based on our spectroscopic data. However, our structure refinements find three different Cu–O bond lengths, consistent with the scenario described in ref (27).

Returning to the optical spectrum, at significantly higher energy, additional excitations would be expected from bonding orbitals having significant ligand sp^3^ character, commonly referred to as “charge-transfer” bands. We attribute absorptions toward the 200 nm end of our spectrum as the latter charge-transfer excitations, involving transitions out of the highest-energy filled bonding orbitals. The gap in the optical range corresponds to the gap between these bonding orbitals and the nonbonding d_*xy*_, d_*xz*_, and d_*yz*_, whose excitations appear at and beyond the infrared end of our spectrum. Previous studies on this and similar Cu(H_2_O)_6_^2+^ compounds have found d–d excitations exclusively in the infrared, starting at ∼850 nm.^28,29^

### 3.1 Magnetization

**Figure 6:**
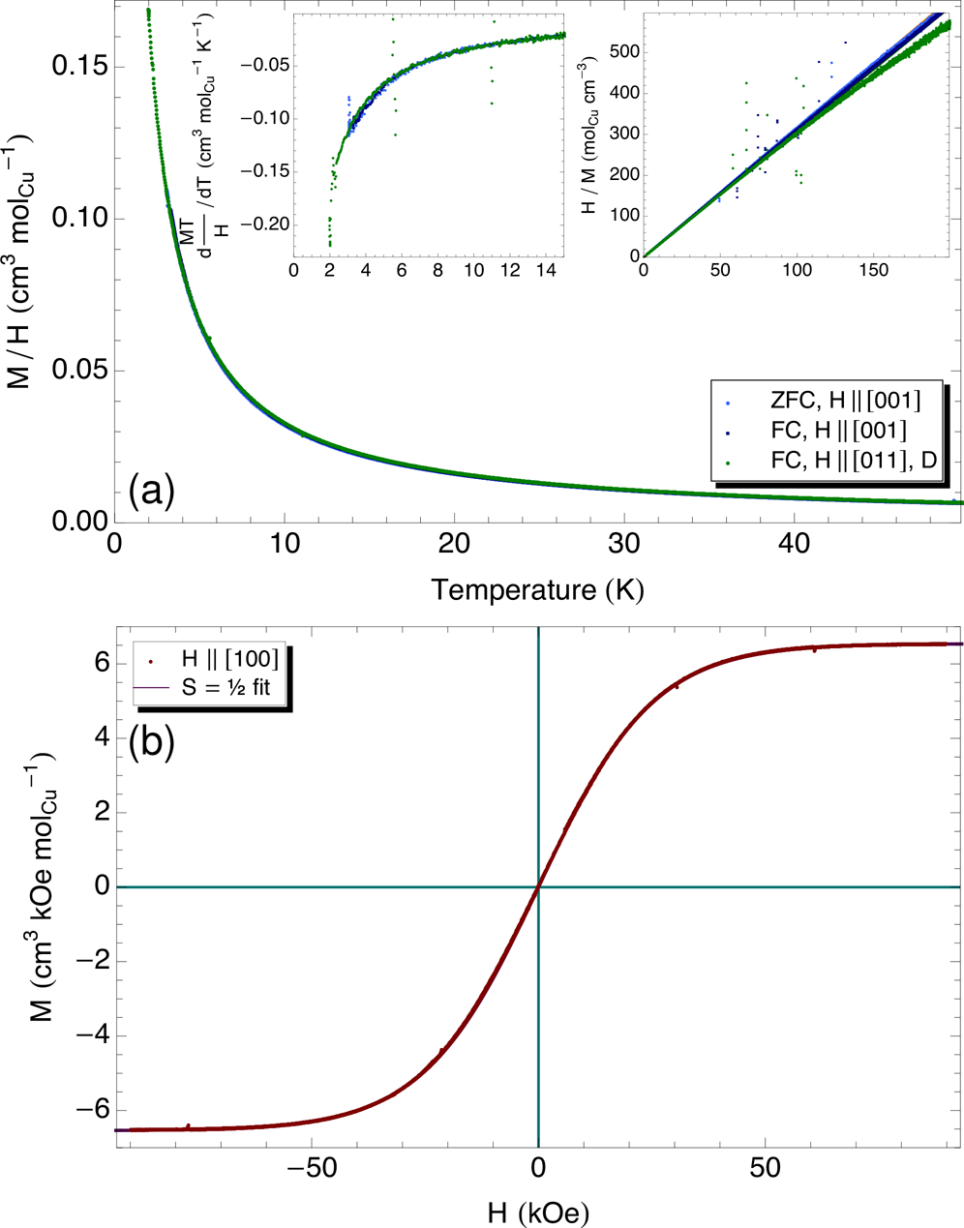
Magnetization of K_2_Cu(SO_4_)_2_·6H_2_O (cyanochroite). (a) Temperature-dependent magnetization under a 1000 Oe field *H*∥[001] and *H*∥[011] in a deuterated crystal shows Curie–Weiss behavior (right inset), with Curie–Weiss temperatures ranging from 33 to 205 mK. The left inset shows the derivative of the magnetization data, with no peak that could suggest a magnetic phase transition. (b) *M*–*H* loop at 2 K for field along [100], fit to the behavior expected for noninteracting *S* = 1/2 spins with only the electron *g* factor as a free parameter.

Magnetization measurements in fields applied along selected directions for both protonated and deuterated crystals are shown in Figure 6. No anisotropy is observed. The temperature-dependent data are well-described below 100 K by a Curie–Weiss law with an offset, i.e., *M*/*H* = *A* + *B*/(*T* – Θ_CW_), as shown in the right inset. The Curie–Weiss temperatures extracted from the zero-field-cooled (ZFC) and field-cooled (FC) data range from 33 to 205 mK, and the paramagnetic moments range from 1.59 to 1.62 μ_B_. No evidence for a magnetic transition is observable in these data or their derivative (left inset), consistent with the extremely small Curie–Weiss temperatures. The slightly positive Θ_CW_ suggests a tendency toward ferromagnetism. Earlier ac susceptometry measurements to lower temperature found a Curie–Weiss temperature of 31.3 mK,^9^ with our higher values likely due to our higher measurement field, and a slightly higher paramagnetic moment of 1.91 μ_B_.

Field-dependent magnetization is shown in Figure 6b. As expected, no hysteresis is observed. The curve is well-described by that of free *S* = 1/2 spins, i.e., *M* = −*N*_A_*nSg*μ_B_ tanh(−*Sg*μ_B_*B*/*k*_B_*T*), where there is *n* = 1 Cu spin per formula unit and *S* = 1/2, with only the electron *g* factor as an adjustable parameter. The *g* factor extracted from this fit is 2.34, suggesting an orbital component, as proposed previously.^9^ The argument of the tanh function reaches unity for our 1000 Oe *M*(*T*) measurement field at a temperature of 79 mK, indicating that our Curie–Weiss temperatures reflect the temperature scale at which the moments would align with the applied field for statistical reasons, with no significant contribution due to exchange interactions. This would lead to an apparent ferromagnetic contribution as found in our fits.

The free-spin-like behavior in the temperature range probed and Curie–Weiss temperatures consistent with a near-absence of exchange interactions reflect that the material’s tortuous exchange pathways (see Figure 1b) prevent any significant exchange coupling among the Cu^2+^ moments. The Cu spins in cyanochroite behave as free and noninteracting to a very good approximation well down into the milliKelvin regime.

## 4 Conclusion

We have refined the structure of cyanochroite, K_2_Cu(SO_4_)_2_·6H_2_O, finding hydrogen positions which differ significantly from those previously reported. These positions are key for modeling of the hydrogen-bonding network, the internal electric fields, and their complex interplay with the Cu^2+^ octahedral distortions. Unlike its NH_4_^+^ analogue, K_2_Cu(SO_4_)_2_·6H_2_O retains the same structure upon deuteration. The hydrogen positions are also crucial for modeling the extremely weak exchange interactions coupling the Cu spins in what remains a model system for studying essentially noninteracting quantum (i.e., *S* = 1/2) spins.

## 5 Experimental Section

### 5.1 Materials and Synthesis

Cyanochroite crystals were grown from aqueous solutions. CuSO_4_·5H_2_O (Alfa Aesar, 99%) and K_2_SO_4_ (Grüssing GmbH, 99%) were each fully dissolved in distilled water; these solutions were then mixed in a glass beaker, and the water was allowed to slowly evaporate. In some cases, a watchglass was added as a lid, and the crystals were grown at 60 °C in a convection drying oven or on a hot plate; in other cases, the beaker was left to evaporate at room temperature with no lid. After several days, pale blue crystals several millimeters in size grew on the bottom of the beaker. The growth process proceeded somewhat more quickly at 60 °C due to the more rapid evaporation even with a lid present, but no significant difference was found among crystals grown by these approaches. Deuterated crystals were grown from D_2_O (Acros Organics, 99.8%D) instead of H_2_O, and these growths were performed in a desiccator with silica gel to exclude the replacement of deuterium with protons from the water vapor in air.

Best results were obtained with copper-rich cation ratios, typically Cu/K ∼ 2:1, to control nucleation—mixtures closer to the ideal stoichiometry produced clusters of small crystals. Copper-rich mixtures initially produced small clusters of slow-growing green kaliochalcite crystals, KCu_2_(SO_4_)_2_[(OH)(H_2_O)], which were readily collected at one side of the beaker by swirling the liquid. Segregation of the solid phases was also achieved using a horizontal temperature gradient—kaliochalcite grows predominantly on the hot side, whereas cyanochroite grows on the cold side. Thermal gradients were achieved most effectively by cantilevering the beaker off the edge of a hot plate.

### 5.2 Crystal Structure Determination and Modeling

A roughly 1.5 × 1.5 × 2 mm^3^ single crystal of K_2_Cu(SO_4_)_2_·6H_2_O was measured using the KOALA white-beam neutron Laue diffractometer at the OPAL Research Reactor at ANSTO, in Australia,^30^ with a 3 mm aperture to fully illuminate the sample. Due to a very high signal-to-background ratio, analyzable data can be obtained by this technique without deuteration. Data were collected at room temperature and at 4 K for multiple orientations. Deuterated crystals were also measured to verify that the structure did not change. Image data processing, including indexing, intensity integration, and wavelength distribution normalization, was performed using LaueG.^24^ Crystal structure refinement was carried out using Jana2006.^31^

Room-temperature single-crystal X-ray diffraction was carried out on a deuterated sample using a Bruker-AXS KAPPA APEX II CCD diffractometer with graphite-monochromated Mo Kα X-ray radiation. The Rigaku CrysAlisPro package was used to index the observed Bragg reflections and perform the necessary data reduction.^32^ Various crystallites with dimensions on the order of 100 μm were investigated, and the results were found to be fully reproducible. The structure was solved using the Superflip charge flipping^33^ and EDMA^34^ Fourier peak search approach as implemented in Jana2006.^31,35^

Density functional theory calculations were performed in VASP^26^ using all the van der Waals corrections and functionals available to optimize the structure for comparison with diffraction results.

### 5.3 Magnetization Measurements

Magnetization was measured on both protonated and deuterated crystals as a function of temperature using a Cryogenic Ltd. cryogen-free measurement system using the vibrating-sample magnetometry option in both ZFC warming and FC cooling conditions. Four- or five-quadrant *M*–*H* loops were measured at several temperatures. The single crystals were mounted either to a plastic straw or to a plastic rod sample holder, and temperature-dependent measurements were performed in 1000 Oe fields applied along the [100], [001], and [011] directions.

### 5.4 UV–Vis Spectroscopy

Absorption spectra in the ultraviolet, visible, and near-infrared range from 200 to 800 nm were collected at room temperature on a Varian Cary 4000 spectrophotometer with a scan rate of 600 nm/min, a step of 1 nm, a spectral bandwidth of 2 nm, and averaging time of 0.1 s. Fifteen milligrams of cyanochroite crystal was ground together with 200 mg of BaSO_4_ to avoid excessive absorption.

## Footnotes

a Most Tutton salts are best known in low-temperature physics or as minerals, and we write the chemical formula using the convention common in these fields. Since the H_2_O groups are coordinated as ligands, the Tutton salts are more accurately described as A_2_[M(H_2_O)_6_](XO_4_)_2_.
